# Novel necroptosis-related gene signature for predicting the prognosis of pancreatic adenocarcinoma

**DOI:** 10.18632/aging.203846

**Published:** 2022-01-24

**Authors:** Zixuan Wu, Xuyan Huang, Minjie Cai, Peidong Huang, Zunhui Guan

**Affiliations:** 1 Guangzhou University of Chinese Medicine, Guangzhou, Guangdong Province 510006, China; 2 Shantou Health School, Shantou, Guangdong Province 515061, China; 3 Yunnan University of Chinese Medicine, Kunming, Yunnan Province 650500, China; 4 Kunming Municipal Hospital of Traditional Chinese Medicine, Kunming, Yunnan Province 650011, China

**Keywords:** pancreatic adenocarcinoma (PAAD), necroptosis-related genes (NRGs), TCGA and GEO datasets, immunity, m6A and immune checkpoint, bioinformatics analysis

## Abstract

Pancreatic adenocarcinoma (PAAD) is a deadly digestive system tumor with a poor prognosis. Recently, necroptosis has been considered as a type of inflammatory programmed cell death. However, the expression of necroptosis-related genes (NRGs) in PAAD and their associations with prognosis remain unclear. NRGs’ prediction potential in PAAD samples from The TCGA and GEO datasets was investigated. The prediction model was constructed using Lasso regression. Co-expression analysis showed that gene expression was closely related to necroptosis. NRGs were shown to be somewhat overexpressed in high-risk people even when no other clinical symptoms were present, indicating that they may be utilized in a model to predict PAAD prognosis. GSEA showed immunological and tumor-related pathways in the high-risk group. Based on the findings, immune function and m6A genes differ significantly between the low-risk and high-risk groups. MET, AM25C, MROH9, MYEOV, FAM111B, Y6D, and PPP2R3A might be related to the oncology process for PAAD patients. Moreover, CASKIN2, TLE2, USP20, SPRN, ARSG, MIR106B, and MIR98 might be associated with low-risk patients with PAAD. NRGs and the relationship of the immune function, immune checkpoints, and m6A gene expression with NRGs in PAAD may be considered as potential therapeutic targets that should be further studied.

## INTRODUCTION

Pancreatic adenocarcinoma (PAAD) is considered as a fatal gastrointestinal tumor globally, with a death rate that is comparable to its incidence [1]. Surgical resection is the only drastic therapy, but the prognosis is dismal. Primary screening of high-risk factors of PAAD has no standard. By contrast, CT, MRI, positron emission tomography/computed tomography is utilized to diagnose PAAD [2]. However, most patients with PPAD are already in advanced stages, and they have missed the opportunity for surgical treatment after being diagnosed. The curative impact of radiation and chemotherapy for PAAD is not precise [3]. Considering that molecularly targeted therapy has become an indispensable method of treating malignant tumors, identifying novel therapeutic targets is critical.

Apoptosis resistance is a significant barrier that causes chemotherapy to fail during cancer treatment. Bypassing the apoptotic pathway to increase cancer cell death can be performed to address this problem [4, 5]. When apoptosis cannot occur properly, the cell will die. Necrosis is initiated as a “substitute” for apoptosis [6]. It is a caspase-independent, regulated necrotic cell death mechanism primarily mediated by receptor-interacting Protein 1 (RIP1), RIP3, and mixed lineage kinase domain-like protein (MLKL) [7]. Necrotic cells will expel their contents, stimulating the inflammatory response of the surrounding cells and activating body’s immunological response. Consequently, cell necrosis plays a significant role in tumorigenesis, metastasis, and infectious and inflammatory disorders [8, 9]. Necroptosis promotes cancer spread, although it could suppress cancer [10–12]. However, only a few sequence-based studies on aberrant gene expression and its relationship with overall survival (OS) in PAAD patients with necroptosis have been conducted.

Immune checkpoint-related gene profiles in patients with PAAD may be used to identify, evaluate, and predict treatment responses [13]. Despite little analysis conducted on the link between NRGs and PAAD, studying the interaction between NRGs, immunity, immunological checkpoints, and m6A with PAAD clinicopathological tumor options is important. The cause and mechanism of PAAD’s abnormal gene expression and necroptosis are unknown. Further research on the altered transcription of NRGs in patients with PAAD is required to investigate the influence of the NRGs pathway on the prognosis of patients with PAAD. Therefore, understanding the impact of NRGs on PAAD development may find a biomarker that might be utilized as a therapeutic target.

**Figure 1:**
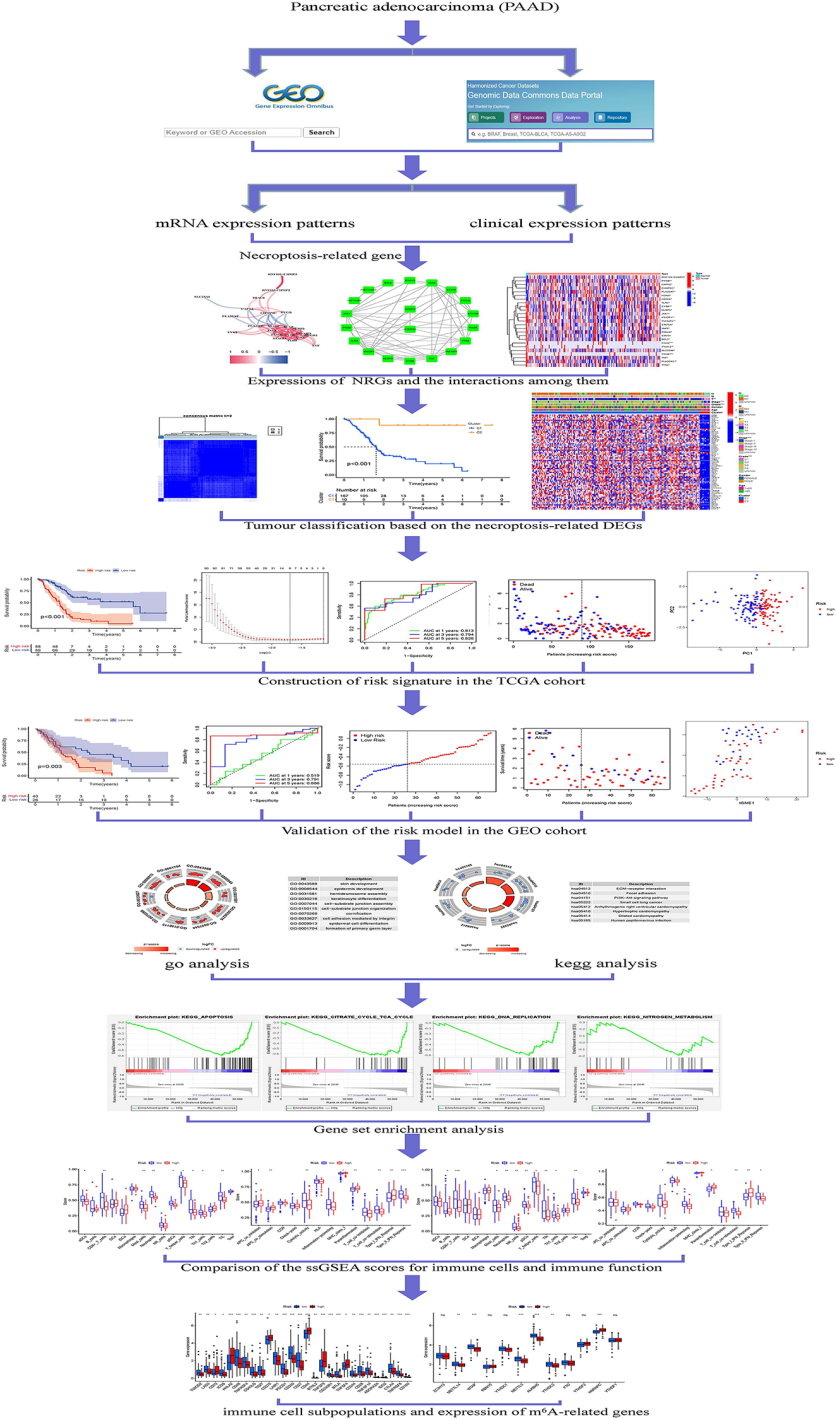
Framework based on an integration strategy of NRGs.

This study aimed to form a prognostic model for PAAD prognosis by spotting NRGs expression related to PAAD patient prognosis. Comprehensively understanding the invasion of NRGs and their associated targets, innovative PAAD therapeutic targets and pharmacologic approaches will be developed. The strategy of NRGs is shown in Figure 1.

## MATERIALS AND METHODS

We followed the methods of Ying Ye et al. 2021 [14].

### Datasets and NRGs

PAAD gene expression patterns and clinical data were collected from the Cancer Genome Atlas (TCGA) [15]. In September 30, 2021, the data of 181 PAAD and 4 normal tissues were enrolled in the TCGA. The Gene Expression Omnibus (GEO) was searched for micro data on mRNA expression. Series: GSE62452. Platform: GPL6244. The GEO shared database was used to maintain the expression patterns of 130 PAAD cases (Table 1). In addition, 52 NRGs were identified from KEGG (https://www.kegg.jp/kegg/) (Supplementary Table 1).

**Table 1:** Clinical characteristics.

**TCGA**		**GEO**	
**Variables**	**Number of samples**	**Variables**	**Number of samples**
Gender		Gender	
Male/Female	102/83	Male/Female	Unknown
Age at diagnosis		Age at diagnosis	
≤65/>65	96/89	≤65/>65	Unknown
Grade		Grade	
G1/G2/G3/G4/NA	32/97/51/2/3	G1/G2/G3/G4/NA	3/64/59/2/2
Stage		Stage	
I/II/III/IV/NA	21/152/4/5/3	I/II/III/IV/NA	7/84/26/13/6
T		T	
T1/T2/T3/T4/NA	7/24/148/4/2	T1/T2/T3/T4	Unknown
M		M	
M0/M1/NA	85/5/95	M0/M1/NA	Unknown
N		N	
N0/N1/N2	80/130/5	N0/N1/N2/N3	Unknown

### Annotation of genes, identification of NRGs and its mutation rates

Transcription and human configuration data were matched by Perl to obtain the precise mRNA gene expression data. The gene IDs were transformed into gene names by R4.1.0 [16]. In order to evaluate the difference of NRGs expression (DEGs) with statistical significance, FDR <0.05 and |log2FC|≥0.585 as a selection criteria. DEG mutation rates were examined using Cbioportal [17] (http://www.cbioportal.org/).

### Tumor classification based on DEGs

First, prognosis-related NRGs were classified into two groups: cluster 1 and 2. Survminer and survival were used to explore the survival and predictive value of PRG subtypes. pheatmap was used to construct a heatmap showing the differential expression and the relationship between NRGs and clinicopathological features of NRGs in each cluster. Limma and corrplot were used to explore the gene connection between PAAD target genes and prognostic NRGs.

### Development of NRGs prognostic signature

The DEGs were split into two classes that supported the median score: low-and high-risk. Lasso regression was related to two classes, and the boldness interval and risk ratio were computed. Survival curves for the two groups were generated and compared. timeROC was used to provide a comparable receiver-operating characteristic (ROC) curve to evaluate the accuracy of this model for predicting survival in PAAD. For the chance curve bestowed by the risk score, NRGs’ risk and survival status were examined. The relationship between clinical characteristics and risk-model was determined, and a similar relationship was found between two NRGs patients. Analyses of risk and clinical relationships are distributed. In addition, investigation was performed using principal component analysis (PCA) and T-distributed neighbor embedding (T-SNE) to analyze whether the prognostic model might properly categorize patients into two risk teams [18]. Desegregating the prognosticative signals, a representation was developed to predict 1-, 3-, and 5-year OS of patients with PAAD.

### Functional enrichment of differentially expressed NRGs

The biological pathways associated with the TCGA DEGs were then examined using Gene Ontology (GO). Biological processes (BP), molecular functions (MF), and cellular components (CC) are controlled by differentially expressed NRGs. NRGs were further investigated using R based on KEGG data [19].

### GSEA enrichment analyses and predictive nomogram

GSEA was used to find related functions and pathway variations. The associated score and graphs were used to verify whether the functions and routes within different risk groups were dynamic. Every sample was classified as “H” or “L” based on whether it had been a high-risk cluster of prognosis-related NRGs.

### Comparison of immune activity among subgroups

Analysis of single-sample sequence set enrichment was utilized (ssGSEA). The enrichment score of immune cells and immune-related activities in two groups was examined in each TCGA and GEO cohort. In addition, the connection among NRGs, checkpoints, and m6A were investigated because these NRGs had significant therapeutic implications.

### Data availability

Patients who granted informed consent to use their data have been uploaded to the public-accessible TCGA and GEO databases.

### Ethics approval and consent to participate

This manuscript is not a clinical trial; hence, ethics approval and consent to participate is not applicable.

## RESULTS

### Differentially expressed NRGs

**Figure 2:**
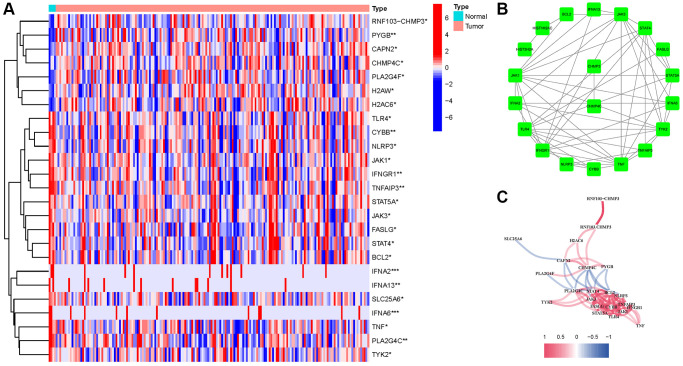
**Expressions of the 25 NRGs and their interactions.** (**A**) Heatmap. (**B**) PPI network. (**C**) Correlation network (red: positive; blue: negative).

**Figure 3:**
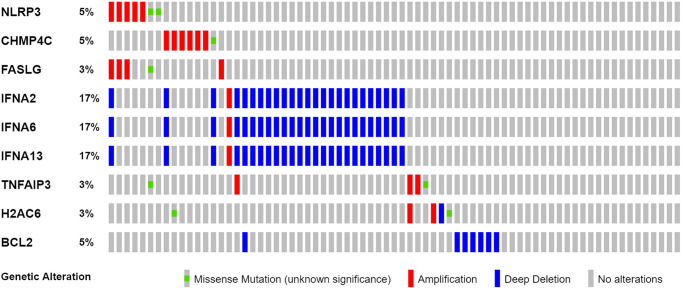
Mutations in NRGs (Abbreviations: N: normal; T: tumor).

We found 25 DEGs related to TCGA (7 upregulated, 18 downregulated; Figure 2A, Supplementary Table 2). We performed a protein–protein interaction (PPI) study to investigate NRGs’ interactions, and the findings are presented in Figure 2B. We discovered that JAK1, TNF, JAK3, IFNGR1, TLR4, and TYK2 were hub genes by setting the low necessary interaction value to 0.4. (Supplementary Table 3). These genes, which included all DEGs discovered in normal and malignant tissues, may be used to determine independent PAAD prognostic markers. Figure 2C demonstrates the correlation network, which includes all NRGs. We investigated genetic changes in these NRGs because of their important clinical implications. We found that the two common mutations were truncating and missense mutations (Figure 3). A total of 9 genes had a 3% mutation rate, with IFNA2, IFNA6, and IFNA13 being the commonly altered (17%).

### Tumor classification based on the DEGs

**Figure 4:**
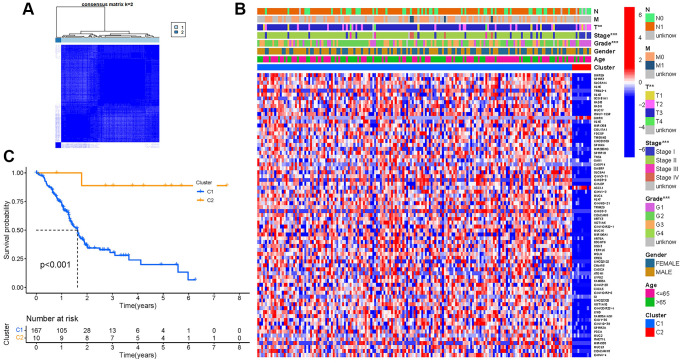
**Tumor classification.** (**A**) Consensus clustering matrix (*k* = 2). (**B**) Heatmap. (**C**) Kaplan–Meier OS curves.

According to Consensus clustering analysis, when the clustering variable (k) was set to 2, the intragroup correlations were the highest, and the intergroup correlations were the lowest, indicating that the 181 PAAD patients could be separated into two groups (Figure 4A). Consequently, DEGs were divided into two clusters: cluster 1 and 2. The gene expression profile and clinical features were shown using a heatmap (Figure 4B). Survival research was conducted to evaluate the prognostic value of NRGs, and the survival rate of cluster 2 was higher than that of cluster 1 (*P* < 0.001, Figure 4C).

### Development of a prognostic gene model in the TCGA cohort

**Figure 5:**
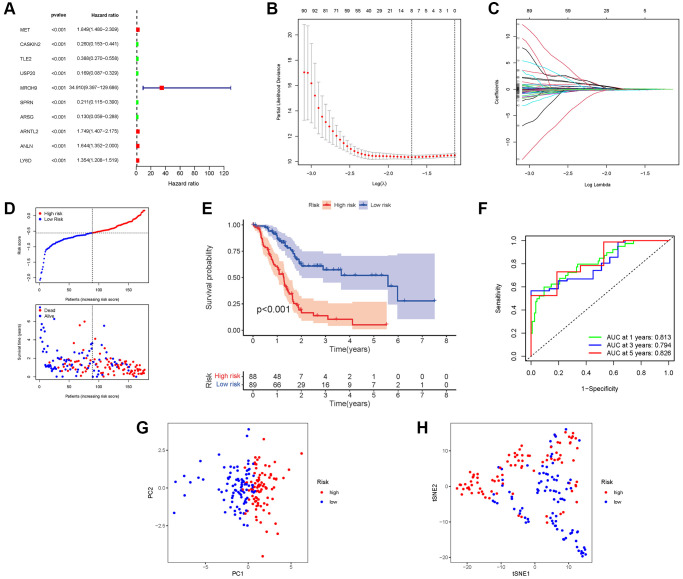
**Construction of risk signature in the TCGA cohort.** (**A**) Univariate cox regression analysis of OS. (**B**) LASSO regression of OS-related genes. (**C**) Cross-validation for tuning the parameter selection. (**D**) Risk survival status plot. (**E**) Kaplan–Meier curve result. (**F**) The AUC of the prediction of 1, 3, 5-year survival rate of PAAD. (**G**) PCA plot. (**H**) t-SNE plot.

We incorporate the TCGA cohort in the training group and the GEO Cohort in the test group to improve the accuracy of the prognostic model. The university COX study identified 10 significant NRGs, which were then included in multivariate COX analysis. A total of 10 NRGs were identified as independent PAAD prognostic markers (MET, CASKIN2, TLE2, USP20, MROH9, SPRN, ARSG, ARNTL2, ANLN, LY6D; Figure 5A). A gene signature was created using the absolute minimal shrinkage and selection operator (LASSO) Cox regression analysis and optimal value (Figure 5B, 5C). We discovered that the risk score of patients was negatively connected to the survival of patients with PAAD. Most of the new NRGs discovered in this investigation showed a negative relationship with the risk model, indicating that additional research is necessary (Figure 5D). Based on Kaplan–Meier analysis, the presence of high-risk PRG signatures was linked with a decreased chance of survival (*P* < 0.001, Figure 5E). For 1, 3, and 5-year survival rates, the AUC predictive value of the unique NRGs signature was 0.813, 0.794, and 0.826, respectively (Figure 5F). The PCA and t-SNE results showed that patients with varying risks were divided into two groups (Figure 5G, 5H).

### External validation of the risk signature

**Figure 6:**
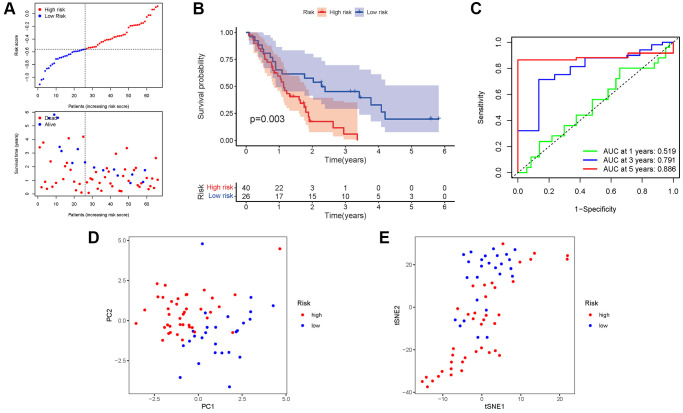
**Validation of the risk model in the GEO cohort.** (**A**) Risk survival status plot. (**B**) Kaplan–Meier curve result. (**C**) The AUC of the prediction of 1, 3, 5-year survival rate of PAAD. (**D**) PCA plot. (**E**) t-SNE plot.

A GEO cohort of 130 PAAD patients served as the validation group. We discovered that patient’s risk score was negatively related to the survival of patients with PAAD. Similar to the TCGA findings, most of the novel NRGs discovered in this investigation were adversely linked with the risk model (Figure 6A). The presence of high-risk PRG signatures was associated with a lower likelihood of survival (*P* = 0.003). Kaplan-Meier analysis was utilized to create Figure 6B. The AUC predictive value of the unique NRGs signature was 0.519, 0.791, and 0.886 for 1, 3, and 5-year survival rates, respectively (Figure 6C). The results of PCA and t-SNE revealed that patients with varied risks were well divided into two groups (Figure 6D, 6E).

### Independent prognostic value of the risk model

**Figure 7:**
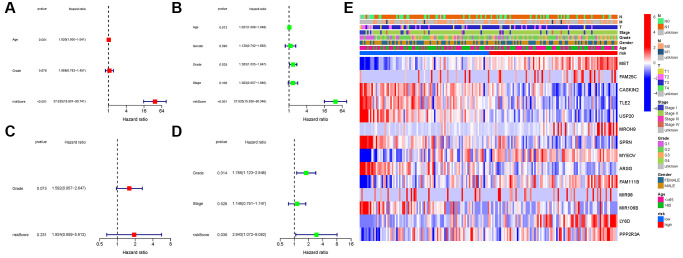
**Univariate and multivariate cox regression analyses.** (**A**, **B**) TCGA cohort. (**C**, **D**) GEO cohort. (**A**, **C**) Univariate analysis. (**B**, **D**) Multivariate analysis. (**E**) Heatmap.

COX analysis in the TCGA cohort revealed that the NRGs signature (HR: 37.625, 95CI: 15.601-90.741) was the primary independent predictor of OS of patients with PAAD (HR: 37.625, 95CI: 15.601–90.741; Figure 7A, 7B). The COX analysis result was demonstrated in the GEO cohort (Figure 7C, 7D). In addition, we created a heatmap of clinical features for the TCGA cohort (Figure 7E, Supplementary Table 4).

### Enrichment analysis of necroptosis-related genes

**Figure 8:**
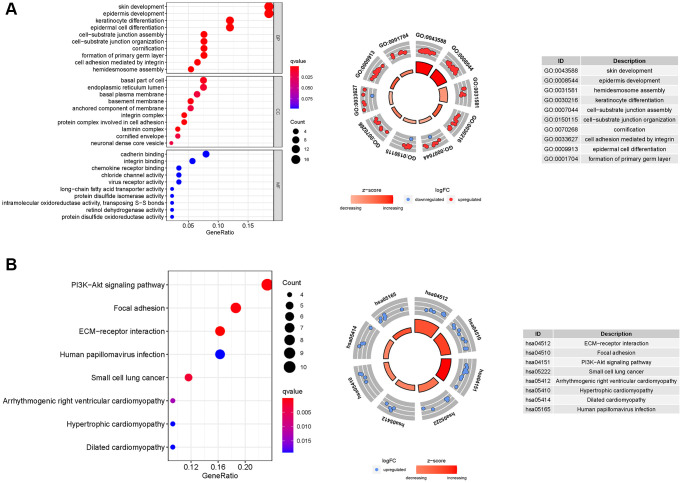
**GO and KEGG analyses for ARGs.** (**A**) GO, (**B**) KEGG.

GO enrichment analysis revealed 83 core targets, including CC and BP. The CC primarily involves the cell–substrate junction (GO:0030055), endoplasmic reticulum lumen (GO:0005788), and basal part of cell (GO:0045178). The BP primarily involves ameboidal-type cell migration (GO:0001667), epidermis development (GO:0008544), epithelial cell proliferation (GO:0050673), and cell junction assembly (GO:0034329, Supplementary Table 5A). In addition, the main signaling pathways were identified by KEGG enrichment analysis, revealing that the over-expressed genes were primarily involved in the PI3K-Akt signaling pathway (hsa04151), focal adhesion (hsa04510), dilated cardiomyopathy (hsa05414), and small cell lung cancer (hsa05222, Figure 8 and Supplementary Table 5B).

### Gene set enrichment analyses

**Figure 9:**
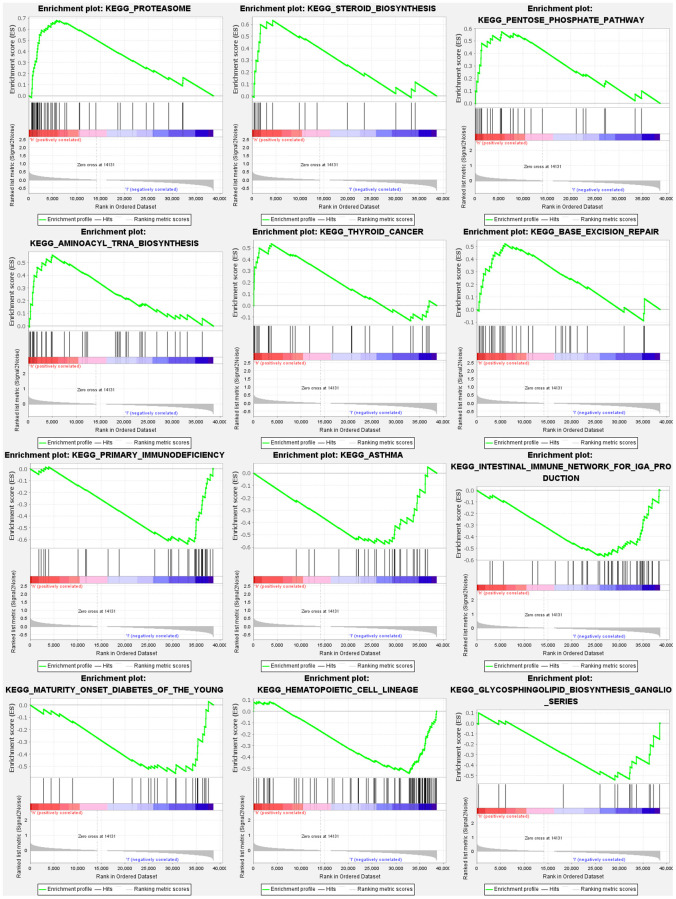
Gene set enrichment analyses.

Based on gene set enrichment analyses (GSEA), the majority of NRGs prognostic signature regulated immune and tumor-related pathways such as proteasome, steroid biosynthesis, pentose phosphate pathway, aminoacyl tRNA biosynthesis, p53, notch, and wnt signaling pathway. The top 6 enriched functions or pathways for each cluster are shown in Figure 9 and Supplementary Table 6A, 6B. Consequently, the “p53 signaling pathway” was the most enriched.

### Comparison of the immune activity among subgroups

**Figure 10:**
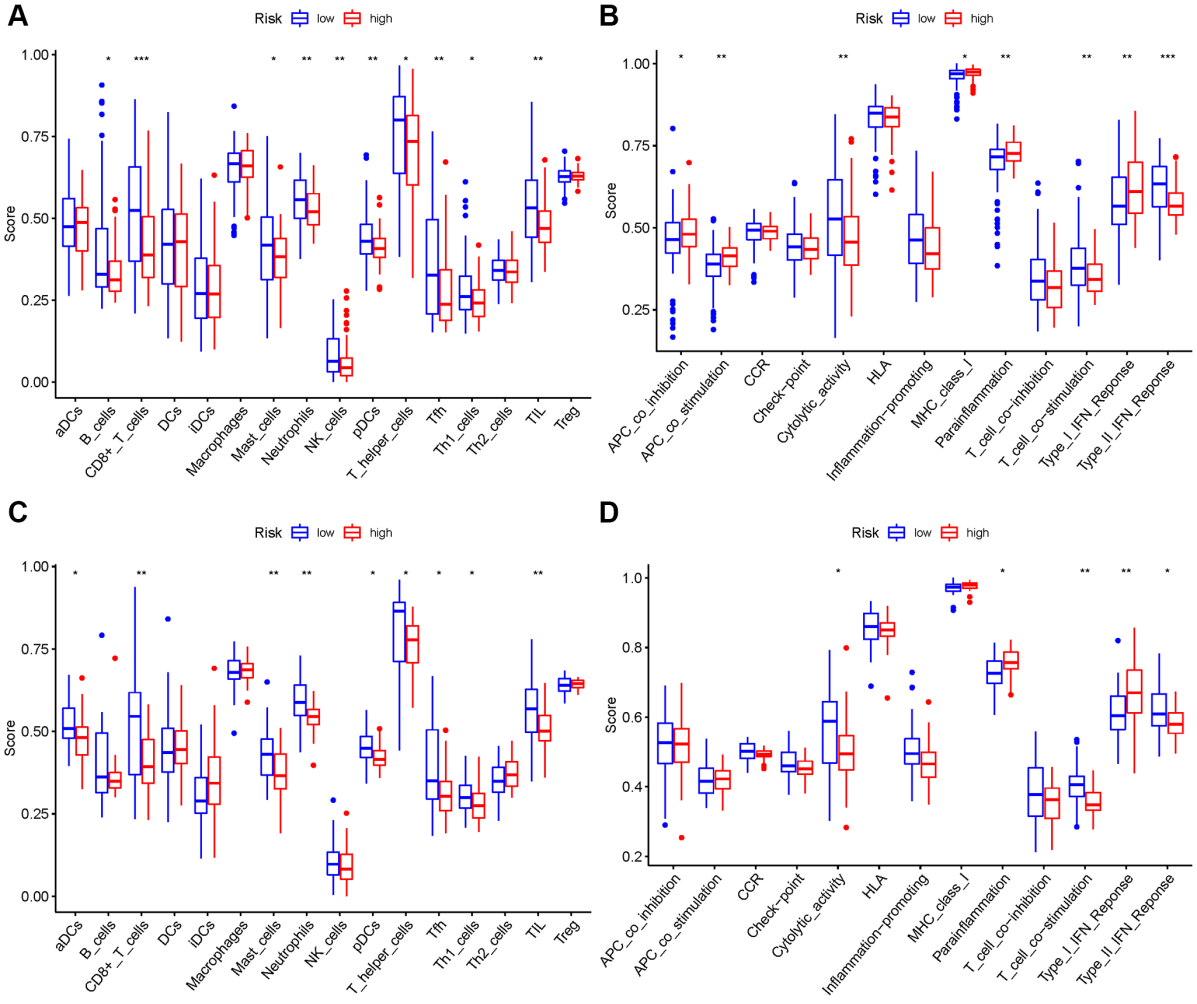
**Comparison among ssGSEA scores.** (**A**, **B**) TCGA cohort, (**C**, **D**) GEO cohort.

We evaluated the enrichment scores of 16 kinds of immune cells and the activity of 13 immune-related functions across low- and high-risk groups (ssGSEA). aDCs, DCs, iDCs, macrophages, Th2 cells, and Treg did not differ substantially between the two groups in the TCGA cohort (*P* > 0.05). Other immune cells infiltrate at a greater rate in the high-risk subgroup (Figure 10A). CCR, check point, HLA, inflammation-related promotion, and T cell co-inhibition were not substantially different between the two groups (*P* > 0.05). Other immune-related functions are usually more significant in the high-risk group (Figure 10B). Similar findings were reached when examining the immunological state of the GEO cohort (Figure 10C, 10D).

### Analysis of the correlation among NRGs with immune checkpoints and m6A

**Figure 11:**
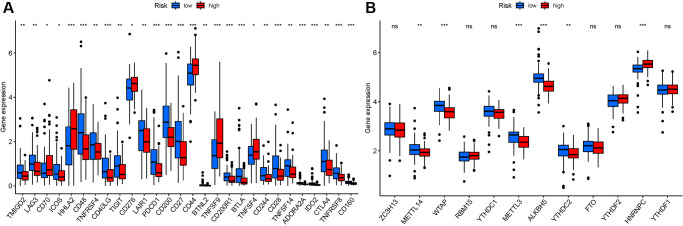
**Analysis of the correlation of NRGs.** (**A**) ICRGs in PAAD risk groups. (**B**) m6A-NRG in PAAD risk groups.

We investigated potential changes in immune checkpoint expression and m6A genes between the two groups. The expression of HHLA2, CD48, CD40LG, PDCD1, CD200, CD27, and other genes differed significantly between the two patient groups (Figure 11A). When the PRG expression was examined between the two-risk groups, METTL3, METTL14, HNRNPC, WTAP, YTHDC2, and ALKBH5 were substantially more significant in the high-risk group (Figure 11B). HNRNPC associated with m6A modification had higher expression in high risk group, suggesting that it might be related to the oncology process for PAAD patients. While METTL14, WTAP, METTL3, ALKBH5, and YTHDC2 had lower expression in high risk group, indicating that they might be tumor suppressor.

## DISCUSSION

Treating PAAD is a severe clinical issue because of its advanced stages and terrible illness [20]. The molecular identification of diagnostic biomarkers and treatment targets for PAAD should always be prioritized. Previous research has shown that vanadium complexes have a selective cytotoxic effect on the human pancreatic ductal adenocarcinoma cell line (PANC-1), causing the mixture of apoptotic and necroptotic processes of PANC-1 cells at increasing doses [21]. Necroptotic programmed cell death is an alternate method of programmed cell death, which can address apoptosis resistance and activate and enhance antitumor immunity in cancer treatment [22]. Necroptosis can serve as a tumor suppressor, making it a potentially practical cancer therapy approach [23]. This research has been conducted to investigate the involvement of essential targets and pathways in PAAD prognosis, resulting in the identification of a feasible biomarker and therapy target.

We found 25 DEGs associated with necroptosis, and the genes were divided into two groups to investigate their possible involvement in PAAD. Based on previous studies, NRGs were significantly linked to PAAD prognosis in a university Cox regression study. The researchers found that 14 prognostic NRGs were expressed differently in risk individuals. Some NRGs were found to be overexpressed in high-risk individuals (*P* < 0.05). In addition, we investigated the role of NRGs in PAAD. A survival study was used to measure the predictive value of NRGs. Patients with low-risk NRGs had longer life span. MET, AM25C, MROH9, MYEOV, FAM111B, Y6D, and PPP2R3A were highly expressed in the high-risk group, indicating that these genes may be related to the oncology process for patients with PAAD, and they seemed to be cancer-promoting genes. The results of the abovementioned genes provide some insights for further research, but conclusive evidence that they are involved in the expression of specific transcription factors related to necroptosis regulation, such as USP22, CDK9, and Foxo1 [24–26] is lacking, which requires further investigation. CASKIN2, TLE2, USP20, SPRN, ARSG, MIR106B, and MIR98 were considered to be substantially expressed in the low-risk group, suggesting that these genes may be PAAD tumor suppressor genes.

DATE truncation activated HGF expression in CRC cell lines, resulting in autocrine signaling via MET, thereby increasing cell proliferation and resistance to necroptosis. HGF signaling via MET decreased the levels of receptor-interacting serine-threonine kinase 1, a necroptosis mediator, in CRC cells [27]. MYEOV promotes pancreatic cancer progression by increasing HES1 expression and SOX9 transactivity [28]. The FAM111B gene mutation is linked to inherited exocrine pancreatic dysfunction [29]. TLE2 is linked to a good prognosis in pancreatic cancer, which regulates cell growth and gemcitabine sensitivity. These studies also demonstrate the validity and credibility of our findings [30]. The OS based on GSE62452 Kaplan-Meier curves and ROC analyses revealed that a necroptosis-related signature might be an independent prognostic predictor. Little research has been conducted on gene changes linked with necroptosis. Therefore, more research is needed to comprehend the mechanism of NRG change and fully identify and corroborate our findings.

In addition, KEGG analysis showed that NRGs were primarily involved in the PI3K-Akt signaling pathway. PI3K enhances tumor necrosis factor-induced necroptosis by activating the RIP1-RIP3-MLKL signaling pathway [31]. Consequently, necroptosis is crucial in PAAD. In GSEA, the p53 signaling pathway was the most significantly enriched pathway. A recent study has shown that p53 not only can cause necrosis via opening the mitochondrial permeability transition pore, but also can interact directly with cyclophilin D (CypD) to open the permeability transition pore (PTP) in the oxidative stress response [32]. *In vitro* and *in vivo* research has revealed that cyclin-dependent kinase inhibitor 3 (CDKN3) is adversely associated with pancreatic cancer tissue prognosis. CDKN3 can form a complex with MDM2-p53 to suppress the production of the P53 target gene P21, promoting the cell cycle and proliferation of PADD cells [33]. Considering the abovementioned criteria, NRGs may influence PAAD cell migration and proliferation via modulating the P53 signaling pathway.

Our method successfully predicts the survival of patients with PAAD. Based on the NRGs prognostic model, a rise in the risk score is associated with an increase in death and high-risk ratio. NRGs may be important biomarkers for predicting outcomes of patients with PAAD. Furthermore, we investigated and studied the relationship among NRGs, immune cells, immunological activity, immune checkpoints, and m6A. Recent research has found a link between various cell death mechanisms and antitumor immunity. Even in ICI-resistant tumors, pyroptosis, ferroptosis, and necroptosis activation combined with ICIs resulted in synergistically increased anticancer efficacy [34, 35]. An immunological checkpoint is the connection between SLC41A3 expression and immune cell invasion [36]. A microscopic investigation of the relationship among ICI, m6A, and pyrolysis has been conducted. Despite little analysis performed on NRGs and PAAD, supported by the abovementioned information, NRGs alterations were associated with the onset and development of PAAD.

Our research has limitations, although it is provided for theoretical underpinnings and research suggestions. First, we were unable to acquire sufficient external data from other publicly available sources to evaluate model’s dependability. Second, we focused on signature of 14 risk-NRGs in our early expression study. However, no further functional or mechanistic research was conducted. Finally, no PAAD studies were conducted to confirm the link between prognostic genes and pyrolysis. Therefore, further research must be conducted to confirm the abovementioned findings.

## CONCLUSIONS

A total of 14 anticipated NRGs were identified in patients with PAAD. The findings contributed to the comprehensive understanding of the relationship among necroptosis, immunological function, ICI, m6A, and immune cells, understanding the potential role of NRGs in the generation and development of PAAD malignant tumors, to provide research ideas for finding new therapeutic targets and prognostic indicators.

## Supplementary Materials

Supplementary Tables
