# Supplementary material for: Novel necroptosis-related gene signature for predicting the prognosis of pancreatic adenocarcinoma
Source: Aging (Albany NY). 2022 Jan 24;14(2):869–91. doi: 10.18632/aging.203846 (PMC8833111; doi:10.18632/aging.203846)
Supplement: Supplementary Tables [file aging-14-203846-s001.pdf]

SUPPLEMENTARY TABLES

Supplementary Table 1. 52 necroptosis-related genes.

BAK1	CHMP4B	IL18	CASP9	NLRP6
BAX	CHMP4C	IL1A	GPX4	NLRP7
CASP1	CHMP6	IL1B	GSDMA	NOD1
CASP3	CHMP7	IRF1	GSDMB	NOD2
CASP4	CYCS	IRF2	GSDMC	PJVK
CASP5	ELANE	TP53	IL6	PLCG1
CHMP2A	GSDMD	TP63	NLRC4	PRKACA
CHMP2B	GSDME	AIM2	NLRP1	PYCARD
CHMP3	GZMB	CASP6	NLRP2	SCAF11
CHMP4A	HMGB1	CASP8	NLRP3	TIRAP
TNF	GZMA			

Supplementary Table 2. 25 DEGs linked to NRGs.

gene	conMean	treatMean	logFC	*p* Value
TNF	2.9597148	0.878954778	−1.751597318	0.023229673
CYBB	44.8476025	13.38835502	−1.74405215	0.004999667
SLC25A6	260.2667	212.1314808	−0.295032001	0.029730049
PYGB	26.3985825	75.0233836	1.506879866	0.002884394
PLA2G4C	3.36357325	1.527573251	−1.138753114	0.007311847
PLA2G4F	0.096944548	0.69805447	2.848107951	0.01144699
CAPN2	29.2810825	50.60433046	0.789291959	0.012420784
NLRP3	2.307436	1.149735351	−1.004988816	0.014207465
RNF103-CHMP3	0.03308378	0.125159531	1.919572176	0.049686083
CHMP4C	7.25279525	14.30096114	0.979503084	0.038630253
FASLG	1.15240325	0.348416184	−1.725762088	0.037736936
IFNA2	0.012599015	0.001135769	−3.471569966	0.000288726
IFNA6	0.021787193	0.001020912	−4.415549216	0.000203649
IFNA13	0.00733201	0.001187969	−2.625711059	0.001901453
IFNGR1	67.564385	40.74547212	−0.729623216	0.009433631
JAK1	49.8170925	34.40897001	−0.533856116	0.044375834
JAK3	12.6223675	4.252687198	−1.569535885	0.019953735
TYK2	19.87963	12.60590665	−0.657191021	0.048594059
STAT4	3.619687	1.434699334	−1.335116524	0.03600294
STAT5A	14.5681125	9.021417197	−0.691387976	0.017539418
TLR4	7.0368955	3.415832457	−1.042701875	0.021537533
TNFAIP3	30.580245	13.05847187	−1.227613886	0.002033049
H2AW	3.70185	10.68664761	1.529491009	0.024420867
H2AC6	10.024392	27.38610677	1.449929449	0.015385115
BCL2	7.155259	2.040894169	−1.809802617	0.014983353

Supplementary Table 3. Hub genes.

Name	Betweenness	Closeness	Degree	Network
JAK1	58.62251082	0.296875	11	9.223809524
TNF	30.05974026	0.287878788	11	9.555555556
JAK3	42.26688312	0.292307692	10	8.103174603
IFNGR1	20.94393939	0.28358209	10	8.158730159
TLR4	32.18744589	0.28358209	10	8.357142857
TYK2	13.49307359	0.28358209	10	8.583333333
STAT4	5.093073593	0.275362319	8	6.726190476
STAT5A	13.33333333	0.271428571	7	5.833333333
IFNA2	0.6	0.263888889	6	5.8
IFNA6	0	0.256756757	5	5
TNFAIP3	1.533333333	0.25	5	4.166666667
CYBB	1.2	0.25	4	3.333333333
NLRP3	0.666666667	0.243589744	4	3.333333333
BCL2	60	0.253333333	3	1
IFNA13	0	0.246753247	3	3
HIST1H2AC	32	0.213483146	2	0
FASLG	0	0.234567901	2	2
CHMP3	0	0.052631579	1	0
CHMP4C	0	0.052631579	1	0
HIST3H2A	0	0.180952381	1	0

Supplementary Table 4. 14 risk PRGs.

Id	TCGA-3A-A9J0	TCGA-2L-AAQL	TCGA-US-A77E	TCGA-RB-AA9M
MET	5.042611	4.7215981	5.9641271	5.2955498
FAM25C	2.0205815	2.2528105	2.0205815	2.0205815
CASKIN2	4.1749081	3.58593	4.0714195	4.1428019
TLE2	4.1259097	4.624045	3.999072	4.7286858
USP20	3.0161423	3.715024	3.7000295	3.641327
MROH9	1.0486983	0.9140702	0.9140702	0.9350862
SPRN	2.6244162	2.8517601	2.3841645	2.6025939
MYEOV	3.2386553	4.1509198	4.4052511	3.667548
ARSG	2.2785722	2.0642247	2.3422646	2.3341557
FAM111B	2.6997762	1.4177758	1.9956956	2.4969894
MIR98	0.8402696	1.218496	1.1183522	0.8402696
MIR106B	2.7306465	1.4784037	1.4784037	2.6675028
LY6D	1.9197985	2.4401492	2.8759444	5.8630146
PPP2R3A	2.3522622	2.2616753	2.9258627	3.446074
riskScore	−0.847267824	−0.659885519	−0.580465013	−0.643074732
risk	low	low	low	low

Supplementary Table 5A. GO enrichment analysis.

Ontology	Description	BgRatio	*p* value	*q* value
BP	skin development	415/18862	1.49E-11	2.35E-08
BP	epidermis development	463/18862	8.27E-11	6.50E-08
BP	hemidesmosome assembly	12/18862	1.91E-09	9.99E-07
BP	keratinocyte differentiation	302/18862	2.51E-07	9.88E-05
BP	cell-substrate junction assembly	100/18862	5.77E-07	0.000181471
BP	cell-substrate junction organization	106/18862	8.58E-07	0.00021942
BP	cornification	113/18862	1.32E-06	0.00021942
BP	cell adhesion mediated by integrin	72/18862	1.38E-06	0.00021942
BP	epidermal cell differentiation	360/18862	1.42E-06	0.00021942
BP	formation of primary germ layer	115/18862	1.49E-06	0.00021942
BP	keratinization	225/18862	1.53E-06	0.00021942
BP	cell-matrix adhesion	230/18862	1.84E-06	0.000241166
BP	gastrulation	179/18862	2.66E-06	0.000321238
BP	extracellular matrix organization	393/18862	3.31E-06	0.000350118
BP	extracellular structure organization	394/18862	3.39E-06	0.000350118
BP	external encapsulating structure organization	396/18862	3.56E-06	0.000350118
BP	cell-substrate adhesion	359/18862	9.94E-06	0.000919336
BP	mesodermal cell differentiation	32/18862	1.72E-05	0.001499725
BP	mesoderm formation	68/18862	2.02E-05	0.001674835
BP	mesoderm morphogenesis	70/18862	2.33E-05	0.001833235
BP	positive regulation of chemotaxis	139/18862	6.09E-05	0.004395847
BP	epithelial cell migration	357/18862	6.15E-05	0.004395847
BP	epithelium migration	360/18862	6.56E-05	0.004483738
BP	negative regulation of anoikis	17/18862	7.27E-05	0.004584778
BP	tissue migration	365/18862	7.29E-05	0.004584778
BP	keratinocyte proliferation	48/18862	8.75E-05	0.005293053
BP	ameboidal-type cell migration	473/18862	0.000102639	0.005822652
BP	entry into host	153/18862	0.00010367	0.005822652
BP	integrin-mediated signaling pathway	106/18862	0.000170071	0.009222742
BP	response to prostaglandin E	23/18862	0.000185292	0.009713186
BP	establishment of skin barrier	24/18862	0.000211015	0.010283511
BP	regulation of anoikis	24/18862	0.000211015	0.010283511
BP	movement in host environment	175/18862	0.000215789	0.010283511
BP	cell junction assembly	425/18862	0.00022915	0.010599087
BP	regulation of water loss via skin	26/18862	0.00026916	0.012093978
BP	positive regulation of cell projection organization	344/18862	0.000277513	0.012122947
BP	positive regulation of granulocyte chemotaxis	27/18862	0.000301738	0.012824934
BP	mesoderm development	123/18862	0.0003398	0.014062637
BP	response to prostaglandin	30/18862	0.000414413	0.016710737
BP	anoikis	34/18862	0.000602246	0.023677781
BP	biological process involved in interaction with host	219/18862	0.000710972	0.027270667
BP	cell chemotaxis	306/18862	0.000745332	0.027907915
BP	regulation of keratinocyte proliferation	37/18862	0.000773772	0.028299038
BP	regulation of chemotaxis	224/18862	0.000799698	0.028582522
BP	bone remodeling	89/18862	0.000943054	0.03295725
BP	regulation of dopaminergic neuron differentiation	10/18862	0.001032363	0.035294066
BP	epithelial cell proliferation	428/18862	0.00116202	0.038038774
BP	regulation of dendritic cell antigen processing and presentation	11/18862	0.001257775	0.038038774
BP	protein folding in endoplasmic reticulum	11/18862	0.001257775	0.038038774
BP	skin morphogenesis	11/18862	0.001257775	0.038038774
BP	dendritic cell apoptotic process	11/18862	0.001257775	0.038038774
BP	regulation of dendritic cell apoptotic process	11/18862	0.001257775	0.038038774
BP	endodermal cell differentiation	44/18862	0.001286801	0.038182347

BP	brown fat cell differentiation	46/18862	0.001464569	0.04265236
BP	establishment of T cell polarity	12/18862	0.001504544	0.043019868
BP	regulation of cell adhesion mediated by integrin	48/18862	0.001657031	0.04570258
BP	tissue homeostasis	260/18862	0.001717022	0.04570258
BP	regulation of granulocyte chemotaxis	49/18862	0.001758907	0.04570258
BP	establishment of lymphocyte polarity	13/18862	0.001772461	0.04570258
BP	immunological synapse formation	13/18862	0.001772461	0.04570258
BP	regulation of insulin secretion	178/18862	0.001794325	0.04570258
BP	positive regulation of plasma membrane bounded cell projection assembly	106/18862	0.001801795	0.04570258
BP	regulation of bone remodeling	51/18862	0.001974208	0.049280987
CC	integrin complex	31/19520	1.38E-05	0.000958944
CC	laminin complex	12/19520	2.23E-05	0.000958944
CC	protein complex involved in cell adhesion	36/19520	2.53E-05	0.000958944
CC	basement membrane	94/19520	8.65E-05	0.002459337
CC	basal part of cell	258/19520	0.000233992	0.005320244
CC	endoplasmic reticulum lumen	306/19520	0.000650914	0.012333107
CC	basal plasma membrane	240/19520	0.001014444	0.016475178
CC	cornified envelope	45/19520	0.001285224	0.016721371
CC	anchored component of membrane	170/19520	0.001323775	0.016721371
CC	neuronal dense core vesicle	13/19520	0.001692709	0.019243428
CC	costamere	18/19520	0.003269266	0.033787629
CC	intermediate filament	215/19520	0.00366975	0.034766056
CC	cell-substrate junction	423/19520	0.004089977	0.035766605
CC	lamellipodium membrane	22/19520	0.004875244	0.039588448
CC	cortical actin cytoskeleton	78/19520	0.006154032	0.045305642
CC	apical plasma membrane	351/19520	0.006659117	0.045305642
CC	microvillus membrane	26/19520	0.006774871	0.045305642
CC	intermediate filament cytoskeleton	256/19520	0.007604382	0.046857122
CC	dense core granule	28/19520	0.007831213	0.046857122
CC	cell-cell junction	485/19520	0.008465165	0.04811778

Supplementary Table 5B. KEGG enrichment analysis.

ID	Description	BgRatio	*p* value	*q* value
hsa04512	ECM-receptor interaction	88/8105	3.28E-07	3.11E-05
hsa04510	Focal adhesion	201/8105	8.56E-06	0.000370605
hsa04151	PI3K-Akt signaling pathway	354/8105	1.17E-05	0.000370605
hsa05222	Small cell lung cancer	92/8105	0.00011567	0.002739557
hsa05412	Arrhythmogenic right ventricular cardiomyopathy	77/8105	0.000701775	0.01329678
hsa05410	Hypertrophic cardiomyopathy	90/8105	0.001260558	0.019074857
hsa05414	Dilated cardiomyopathy	96/8105	0.001601543	0.019074857
hsa05165	Human papillomavirus infection	331/8105	0.001610766	0.019074857

Supplementary Table 6A. GSEA of high risk.

NAME	ES	NES	NOM *p*-val	FDR *q*-val
KEGG_PENTOSE_PHOSPHATE_PATHWAY	0.5729152	1.6310743	0.01953125	1
KEGG_PROTEASOME	0.6784165	1.6303458	0.049701788	0.6821798
KEGG_GLYCOSPHINGOLIPID_BIOSYNTHESIS_LACTO_AND_NEOLACTO_SERIES	0.5090886	1.5907345	0.024952015	0.6408247
KEGG_THYROID_CANCER	0.53489053	1.5439887	0.05179283	0.6907832
KEGG_P53_SIGNALING_PATHWAY	0.47881857	1.5324117	0.04347826	0.60003185
KEGG_GLYCOLYSIS_GLUCONEOGENESIS	0.47841245	1.5322174	0.037698414	0.50075066
KEGG_ADHERENS_JUNCTION	0.4795818	1.5175084	0.0662768	0.47806197
KEGG_STEROID_BIOSYNTHESIS	0.6314558	1.4914162	0.09486166	0.4986435
KEGG_PATHOGENIC_ESCHERICHIA_COLI_INFECTION	0.48299125	1.4645706	0.086105675	0.524178
KEGG_TIGHT_JUNCTION	0.39693546	1.4420464	0.063872255	0.54062873
KEGG_AMINOACYL_TRNA_BIOSYNTHESIS	0.5580433	1.4407	0.11516315	0.49625063
KEGG_STARCH_AND_SUCROSE_METABOLISM	0.46116272	1.4073405	0.08583691	0.5482185
KEGG_GALACTOSE_METABOLISM	0.48794708	1.3951346	0.091617934	0.53853256
KEGG_CELL_CYCLE	0.4647909	1.3540881	0.19960861	0.61424387
KEGG_AXON_GUIDANCE	0.3714874	1.3263278	0.10852713	0.6551905
KEGG_BASE_EXCISION_REPAIR	0.5224427	1.3245112	0.1809145	0.6192906
KEGG_O_GLYCAN_BIOSYNTHESIS	0.45017973	1.321248	0.1764706	0.5923887
KEGG_PORPHYRIN_AND_CHLOROPHYLL_METABOLISM	0.42830938	1.3130741	0.13465346	0.5810344
KEGG_GLYCOSAMINOGLYCAN_BIOSYNTHESIS_KERATAN_SULFATE	0.48650512	1.3112824	0.13765182	0.55493397
KEGG_PENTOSE_AND_GLUCURONATE_INTERCONVERSIONS	0.46371907	1.2722654	0.18218623	0.62946343
KEGG_BLADDER_CANCER	0.39896894	1.256486	0.18992248	0.6436727
KEGG_REGULATION_OF_ACTIN_CYTOSKELETON	0.33413285	1.250563	0.17105263	0.62979627
KEGG_PANCREATIC_CANCER	0.39570457	1.2432532	0.22896282	0.6211923
KEGG_PATHWAYS_IN_CANCER	0.33537713	1.2430347	0.17017208	0.59580684
KEGG_DRUG_METABOLISM_OTHER_ENZYMES	0.40908697	1.2375876	0.1609658	0.58401114
KEGG_SMALL_CELL_LUNG_CANCER	0.3896531	1.2231851	0.22178218	0.59402245
KEGG_ENDOCYTOSIS	0.3299927	1.2194865	0.20610687	0.58074325
KEGG_ONE_CARBON_POOL_BY_FOLATE	0.47091427	1.2165351	0.256167	0.5663879
KEGG_AMINO_SUGAR_AND_NUCLEOTIDE_SUGAR_METABOLISM	0.4007442	1.2126254	0.23745173	0.55516595
KEGG_N_GLYCAN_BIOSYNTHESIS	0.40781587	1.2106607	0.23287672	0.5410943
KEGG_ASCORBATE_AND_ALDARATE_METABOLISM	0.45372823	1.2069958	0.2371134	0.530202
KEGG_DNA_REPLICATION	0.50567645	1.1796668	0.35166994	0.56995404
KEGG_OOCYTE_MEIOSIS	0.36010596	1.1793286	0.26061776	0.55347526
KEGG_SYSTEMIC_LUPUS_ERYTHEMATOSUS	0.37165263	1.1768961	0.25851703	0.54163045
KEGG_FRUCTOSE_AND_MANNOSE_METABOLISM	0.39953077	1.1730746	0.29766536	0.53443813
KEGG_ARRHYTHMOGENIC_RIGHT_VENTRICULAR_CARDIOMYOPATHY_ARVC	0.35322598	1.1730168	0.26061776	0.5197308
KEGG_FOCAL_ADHESION	0.34690592	1.1704245	0.2751938	0.51039374
KEGG_MISMATCH_REPAIR	0.4998743	1.1677153	0.3300199	0.50204176
KEGG_NOTCH_SIGNALING_PATHWAY	0.35844958	1.1651915	0.25581396	0.49362785
KEGG_ECM_RECEPTOR_INTERACTION	0.38675836	1.1465491	0.3151751	0.51444507
KEGG_PYRIMIDINE_METABOLISM	0.35571525	1.1058619	0.34740883	0.5765829
KEGG_STEROID_HORMONE_BIOSYNTHESIS	0.33638063	1.0779973	0.34631148	0.6170261
KEGG_BIOSYNTHESIS_OF_UNSATURATED_FATTY_ACIDS	0.39363536	1.0769395	0.35416666	0.60520357
KEGG_ENDOMETRIAL_CANCER	0.3395971	1.0718687	0.40151516	0.60124856
KEGG_GLUTATHIONE_METABOLISM	0.32758725	1.0697683	0.35	0.5916402
KEGG_HOMOLOGOUS_RECOMBINATION	0.42976084	1.0597887	0.44646466	0.59751785
KEGG_BASAL_CELL_CARCINOMA	0.32655776	1.0506406	0.38491297	0.6011576
KEGG_CHRONIC_MYELOID_LEUKEMIA	0.33301565	1.0484524	0.4165067	0.5925686
KEGG_WNT_SIGNALING_PATHWAY	0.2844261	1.0387905	0.39961758	0.598086
KEGG_NUCLEOTIDE_EXCISION_REPAIR	0.37913045	1.0309824	0.44656488	0.60022134
KEGG_RENAL_CELL_CARCINOMA	0.3186963	1.0266781	0.41917294	0.59651726
KEGG_SPLICEOSOME	0.36476433	1.0200957	0.4743833	0.5970973

KEGG_PROSTATE_CANCER	0.30396676	1.0045717	0.433526	0.6138756
KEGG_METABOLISM_OF_XENOBIOTICS_BY_CYTOCHROME_P450	0.31419396	0.9750271	0.4947589	0.6572137
KEGG_UBIQUITIN_MEDIATED_PROTEOLYSIS	0.30457303	0.9725739	0.4952199	0.6495022
KEGG_APOPTOSIS	0.30550128	0.9666593	0.5116279	0.6480138
KEGG_HYPERTROPHIC_CARDIOMYOPATHY_HCM	0.2806289	0.9614698	0.5190381	0.6459331
KEGG_VEGF_SIGNALING_PATHWAY	0.27282158	0.9604114	0.5095785	0.63673884
KEGG_RNA_DEGRADATION	0.3311516	0.95151705	0.54285717	0.641828
KEGG_ALZHEIMERS_DISEASE	0.30191407	0.9506232	0.5248509	0.632659
KEGG_RETINOL_METABOLISM	0.3078011	0.93862295	0.53503186	0.6427308
KEGG_ETHER_LIPID_METABOLISM	0.2848933	0.92489564	0.5694165	0.65457636
KEGG_MELANOMA	0.25767887	0.9193458	0.5755814	0.65356475
KEGG_COLORECTAL_CANCER	0.28481606	0.9170352	0.54990584	0.64734876
KEGG_CYSTEINE_AND_METHIONINE_METABOLISM	0.2772554	0.91319263	0.5952849	0.6442672
KEGG_PROTEIN_EXPORT	0.3686309	0.89727354	0.59029126	0.65978324
KEGG_DRUG_METABOLISM_CYTOCHROME_P450	0.27977046	0.895435	0.61349696	0.65332603
KEGG_TERPENOID_BACKBONE_BIOSYNTHESIS	0.37947118	0.88096434	0.59100205	0.6673157
KEGG_LONG_TERM_POTENTIATION	0.265575	0.87706816	0.6404715	0.66389406
KEGG_ARGININE_AND_PROLINE_METABOLISM	0.25249606	0.8637797	0.6546906	0.67669934
KEGG_BASAL_TRANSCRIPTION_FACTORS	0.28742987	0.8605561	0.62081784	0.67225385
KEGG_CITRATE_CYCLE_TCA_CYCLE	0.33972847	0.8536729	0.60240966	0.67510945
KEGG_NOD_LIKE_RECEPTOR_SIGNALING_PATHWAY	0.27057242	0.8370338	0.6673077	0.6935009
KEGG_NITROGEN_METABOLISM	0.27890974	0.82567173	0.75390625	0.70317525
KEGG_DORSO_VENTRAL_AXIS_FORMATION	0.28094056	0.8064852	0.73410404	0.72584325
KEGG_TGF_BETA_SIGNALING_PATHWAY	0.24235316	0.7868584	0.71881187	0.7473405
KEGG_HUNTINGTONS_DISEASE	0.24811034	0.77978605	0.6855469	0.7484468
KEGG_PEROXISOME	0.23973116	0.75253385	0.7090559	0.7813006
KEGG_LINOLEIC_ACID_METABOLISM	0.24497381	0.6923127	0.8792757	0.8565561
KEGG_ANTIGEN_PROCESSING_AND_PRESENTATION	0.23718034	0.6906942	0.814	0.84799135
KEGG_OXIDATIVE_PHOSPHORYLATION	0.16179605	0.4302852	0.9700599	0.9947666

Supplementary Table 6B. GSEA of low risk.

NAME	ES	NES	NOM *p*-val	FDR *q*-val
KEGG_NEUROACTIVE_LIGAND_RECEPTOR_INTERACTION	−0.5290096	−1.890673	0	0.07818665
KEGG_TYPE_II_DIABETES_MELLITUS	−0.53263277	−1.7101595	0.003976143	0.41124317
KEGG_CALCIUM_SIGNALING_PATHWAY	−0.47805348	−1.7010419	0	0.3046739
KEGG_TRYPTOPHAN_METABOLISM	−0.5109064	−1.611668	0.0125	0.49959582
KEGG_GLYCINE_SERINE_AND_THREONINE_METABOLISM	−0.5354933	−1.5545669	0.045009784	0.6258049
KEGG_VASCULAR_SMOOTH_MUSCLE_CONTRACTION	−0.45131353	−1.5381501	0.033970278	0.5840838
KEGG_TASTE_TRANSDUCTION	−0.47686335	−1.5074612	0.050632913	0.61628556
KEGG_HEMATOPOIETIC_CELL_LINEAGE	−0.5428187	−1.4974189	0.08730159	0.57271385
KEGG_PRIMARY_IMMUNODEFICIENCY	−0.6386303	−1.4330411	0.18431373	0.75888455
KEGG_JAK_STAT_SIGNALING_PATHWAY	−0.43019027	−1.4310992	0.103658535	0.69109493
KEGG_ABC_TRANSPORTERS	−0.44498968	−1.4232751	0.06841046	0.65386295
KEGG_ADIPOCYTOKINE_SIGNALING_PATHWAY	−0.4124259	−1.4004673	0.07692308	0.6760876
KEGG_PHOSPHATIDYLINOSITOL_SIGNALING_SYSTEM	−0.43768927	−1.3972843	0.09850107	0.634523
KEGG_GLYCOSPHINGOLIPID_BIOSYNTHESIS_GANGLIO_SERIES	−0.54259735	−1.3912437	0.10261569	0.6088296
KEGG_INTESTINAL_IMMUNE_NETWORK_FOR_IGA_PRODUCTION	−0.5721416	−1.3898985	0.17773438	0.5723439
KEGG_MATURITY_ONSET_DIABETES_OF_THE_YOUNG	−0.55656147	−1.3844813	0.14003944	0.5516851
KEGG_CHEMOKINE_SIGNALING_PATHWAY	−0.4294434	−1.3836561	0.12704918	0.52157277
KEGG_ASTHMA	−0.5758721	−1.382636	0.17153996	0.49566385
KEGG_CELL_ADHESION_MOLECULES_CAMS	−0.43577835	−1.362344	0.17540322	0.5201028
KEGG_CYTOKINE_CYTOKINE_RECEPTOR_INTERACTION	−0.40754113	−1.3578213	0.12525667	0.5050087
KEGG_MTOR_SIGNALING_PATHWAY	−0.42019013	−1.3523698	0.106471814	0.49395788
KEGG_REGULATION_OF_AUTOPHAGY	−0.42233485	−1.3461435	0.10816327	0.48515308
KEGG_TYROSINE_METABOLISM	−0.42170665	−1.341936	0.10766046	0.47379693

KEGG_GLYCOSAMINOGLYCAN_DEGRADATION	−0.48054728	−1.3278168	0.15605749	0.48577037
KEGG_FC_EPSILON_RI_SIGNALING_PATHWAY	−0.39328453	−1.3148739	0.15212981	0.4949966
KEGG_PPAR_SIGNALING_PATHWAY	−0.38816133	−1.297917	0.1399177	0.51296306
KEGG_LONG_TERM_DEPRESSION	−0.36285532	-1.2761356	0.13279678	0.5412
KEGG_COMPLEMENT_AND_COAGULATION_CASCADES	−0.3989361	−1.263181	0.16384181	0.5521064
KEGG_TYPE_I_DIABETES_MELLITUS	−0.4928752	−1.2604029	0.26732674	0.53883076
KEGG_PROXIMAL_TUBULE_BICARBONATE_RECLAMATION	−0.44412115	−1.2592758	0.18590999	0.522951
KEGG_PRIMARY_BILE_ACID_BIOSYNTHESIS	−0.49527532	−1.2533015	0.18442623	0.51886904
KEGG_GNRH_SIGNALING_PATHWAY	−0.34357572	−1.2519171	0.14784394	0.50569147
KEGG_INOSITOL_PHOSPHATE_METABOLISM	−0.39771664	−1.2419469	0.21920668	0.51269734
KEGG_MAPK_SIGNALING_PATHWAY	−0.32389942	−1.2344974	0.1764706	0.51308984
KEGG_ALDOSTERONE_REGULATED_SODIUM_REABSORPTION	−0.3750422	−1.22868	0.16359918	0.5105368
KEGG_T_CELL_RECEPTOR_SIGNALING_PATHWAY	−0.411573	−1.2120905	0.28456914	0.5295486
KEGG_GLYCOSAMINOGLYCAN_BIOSYNTHESIS_CHONDROITIN_SULFATE	−0.45656192	−1.206142	0.2774451	0.52710754
KEGG_NICOTINATE_AND_NICOTINAMIDE_METABOLISM	−0.38467285	−1.2003758	0.23868313	0.52426314
KEGG_AUTOIMMUNE_THYROID_DISEASE	−0.43872103	−1.1698861	0.31547618	0.57136863
KEGG_MELANOGENESIS	−0.3255055	−1.1616304	0.23982869	0.57462007
KEGG_ALLOGRAFT_REJECTION	−0.5078549	−1.158935	0.36399218	0.56604004
KEGG_GAP_JUNCTION	−0.3317633	−1.1554563	0.26283368	0.55955046
KEGG_FATTY_ACID_METABOLISM	−0.40320912	−1.1531155	0.29012346	0.5516308
KEGG_SELENOAMINO_ACID_METABOLISM	−0.37339255	−1.1386981	0.30241936	0.56841093
KEGG_BETA_ALANINE_METABOLISM	−0.39741	−1.1361926	0.28252032	0.5608648
KEGG_GLYCEROPHOSPHOLIPID_METABOLISM	−0.31059223	−1.1272689	0.26612905	0.56516355
KEGG_OLFACTORY_TRANSDUCTION	−0.24886155	−1.115168	0.22376238	0.57648504
KEGG_PRION_DISEASES	−0.36823776	−1.1127352	0.3093385	0.56920344
KEGG_INSULIN_SIGNALING_PATHWAY	−0.3109605	−1.1121604	0.3268817	0.5585135
KEGG_BUTANOATE_METABOLISM	−0.37009233	−1.0814086	0.36491936	0.6072234
KEGG_PANTOTHENATE_AND_COA_BIOSYNTHESIS	−0.37421823	−1.0770823	0.3530572	0.6036107
KEGG_CARDIAC_MUSCLE_CONTRACTION	−0.32574925	−1.0768429	0.371134	0.5923154
KEGG_PROGESTERONE_MEDIATED_OOCYTE_MATURATION	−0.32766244	−1.0730956	0.36247334	0.588726
KEGG_VALINE_LEUCINE_AND_ISOLEUCINE_DEGRADATION	−0.37054572	−1.0679052	0.37623763	0.5874844
KEGG_DILATED_CARDIOMYOPATHY	−0.31053647	−1.0668976	0.34879032	0.5784172
KEGG_EPITHELIAL_CELL_SIGNALING_IN_HELICOBACTER_PYLORI_ INFECTION	−0.31845626	−1.0656122	0.36916837	0.5703242
KEGG_GRAFT_VERSUS_HOST_DISEASE	−0.4773792	−1.0647981	0.42629483	0.56208354
KEGG_LYSINE_DEGRADATION	−0.3400369	−1.0595781	0.39591837	0.5619305
KEGG_FC_GAMMA_R_MEDIATED_PHAGOCYTOSIS	−0.32370082	−1.056988	0.3877551	0.55720764
KEGG_GLYCOSAMINOGLYCAN_BIOSYNTHESIS_HEPARAN_SULFATE	−0.36602896	−1.0548011	0.37708333	0.5525173
KEGG_ARACHIDONIC_ACID_METABOLISM	−0.30121955	−1.0340339	0.39314517	0.57759076
KEGG_RIBOFLAVIN_METABOLISM	−0.3628063	−1.0269295	0.41041666	0.58104
KEGG_B_CELL_RECEPTOR_SIGNALING_PATHWAY	−0.35677096	−1.0201818	0.45039684	0.5839937
KEGG_PHENYLALANINE_METABOLISM	−0.3458501	−1.0084561	0.44855967	0.59530926
KEGG_RENIN_ANGIOTENSIN_SYSTEM	−0.39185286	−0.9946408	0.48336595	0.61027443
KEGG_HISTIDINE_METABOLISM	−0.32132426	−0.9890151	0.48033127	0.6116145
KEGG_LYSOSOME	−0.30735183	−0.9859231	0.4526316	0.6080056
KEGG_VIBRIO_CHOLERAE_INFECTION	−0.2991553	−0.9856829	0.4556701	0.59937316
KEGG_PURINE_METABOLISM	−0.271922	−0.98382556	0.46637744	0.5934177
KEGG_RNA_POLYMERASE	−0.34659335	−0.95722973	0.50988144	0.6307953
KEGG_NEUROTROPHIN_SIGNALING_PATHWAY	−0.27906525	−0.92767113	0.56959313	0.67536575
KEGG_ACUTE_MYELOID_LEUKEMIA	−0.30859992	−0.92660505	0.54037267	0.66776955
KEGG_AMYOTROPHIC_LATERAL_SCLEROSIS_ALS	−0.27340278	−0.92612875	0.5708419	0.6596064
KEGG_NON_SMALL_CELL_LUNG_CANCER	−0.30022708	−0.92514	0.55737704	0.65228117
KEGG_LEUKOCYTE_TRANSENDOTHELIAL_MIGRATION	−0.27998474	−0.9202179	0.5386266	0.6523266
KEGG_ERBB_SIGNALING_PATHWAY	−0.26887137	−0.91797024	0.5744235	0.6477343
KEGG_HEDGEHOG_SIGNALING_PATHWAY	−0.28201923	−0.9156276	0.55737704	0.64300805
KEGG_NATURAL_KILLER_CELL_MEDIATED_CYTOTOXICITY	−0.28414455	−0.90748185	0.54969573	0.6490106
KEGG_SPHINGOLIPID_METABOLISM	−0.29363042	−0.9023531	0.60162604	0.6489709

KEGG_PROPANOATE_METABOLISM	−0.3202926	−0.8933692	0.59356135	0.6564102
KEGG_GLIOMA	−0.26588964	−0.87776834	0.6012397	0.67394316
KEGG_GLYCEROLIPID_METABOLISM	−0.25562853	−0.87741315	0.645749	0.66659164
KEGG_OTHER_GLYCAN_DEGRADATION	−0.3497702	−0.8746694	0.5978947	0.6635369
KEGG_LEISHMANIA_INFECTION	−0.31960237	−0.8571332	0.6122449	0.6853992
KEGG_ALANINE_ASPARTATE_AND_GLUTAMATE_METABOLISM	−0.26438203	−0.8277334	0.71398747	0.72542256
KEGG_VIRAL_MYOCARDITIS	−0.27853227	−0.82128996	0.62890625	0.7271384
KEGG_TOLL_LIKE_RECEPTOR_SIGNALING_PATHWAY	−0.25039133	−0.80701625	0.66935486	0.7418337
KEGG_RIG_I_LIKE_RECEPTOR_SIGNALING_PATHWAY	−0.24272378	−0.7962348	0.71666664	0.7515454
KEGG_VASOPRESSIN_REGULATED_WATER_REABSORPTION	−0.257244	−0.7952852	0.7171717	0.7445486
KEGG_SNARE_INTERACTIONS_IN_VESICULAR_TRANSPORT	−0.26335508	−0.79454243	0.71369296	0.7376034
KEGG_PYRUVATE_METABOLISM	−0.25223786	−0.79080826	0.73140496	0.7351275
KEGG_GLYCOSYLPHOSPHATIDYLINOSITOL_GPI_ANCHOR_BIOSYNTHESIS	−0.29210636	−0.7644893	0.72938687	0.76874876
KEGG_ALPHA_LINOLENIC_ACID_METABOLISM	−0.2534912	−0.7415896	0.87829614	0.79504913
KEGG_GLYOXYLATE_AND_DICARBOXYLATE_METABOLISM	−0.29077378	−0.7335219	0.78644764	0.79881746
KEGG_CYTOSOLIC_DNA_SENSING_PATHWAY	−0.23144941	−0.7221297	0.8114754	0.80624986
KEGG_RIBOSOME	−0.3269558	−0.6639908	0.7683168	0.8751104
KEGG_PARKINSONS_DISEASE	−0.18312952	−0.5185772	0.92785573	0.9770979
